# Cardiovascular Collapse after the Induction of Anesthesia Due to the MASS Effect of Unruptured Giant Bullae

**DOI:** 10.3390/medicina59091689

**Published:** 2023-09-20

**Authors:** Junghyun Park, Dulee Kim, Jae-Hoo Park, Ji-Yun Lee, Eun-Jung Cho

**Affiliations:** 1 Department of Anesthesiology and Pain Medicine, Incheon St. Mary’s Hospital, College of Medicine, The Catholic University of Korea, Incheon 21431, Republic of Korea; happyjj@catholic.ac.kr (J.P.); 22000346@cmcnu.or.kr (D.K.); jhp372@cmcnu.or.kr (J.-H.P.); 2 Department of Thoracic and Cardiovascular Surgery, Incheon St. Mary’s Hospital, College of Medicine, The Catholic University of Korea, Incheon 21431, Republic of Korea; 21500412@cmcnu.or.kr

**Keywords:** giant emphysematous bulla, mass effect, cardiovascular collapse, bullectomy

## Abstract

*Background and Objectives*: Giant bullae rupture easily and cause tension pneumothorax, which can cause problems during general anesthesia. However, the hemodynamic instability that can occur due to the mass effect of an unruptured giant bulla should not be overlooked. *Case report*: A 43-year-old male patient visited the emergency room with an abdominal wound. There was a giant emphysematous bulla in the left lung. Emergency surgery was decided upon because there was active bleeding according to abdominal CT. After tracheal intubation, the patient’s blood pressure and pulse rate dramatically decreased. His blood pressure did not recover despite the use of vasopressors and discontinuation of positive pressure ventilation applied to the lungs. Thus, a bullectomy was immediately performed. The patient’s blood pressure and pulse rate were normalized after the bullectomy. *Conclusions*: If emergency surgery under general anesthesia is required in a patient with a giant emphysematous bulla, it is safe to minimize positive pressure ventilation and remove the giant emphysematous bulla as soon as possible before proceeding with the remainder of the surgery. Tension pneumothorax due to the rupturing of a bulla should be considered first. However, hemodynamic changes might occur due to the mass effect caused by a giant bulla.

## 1. Introduction

A bulla is defined as a cyst with a diameter greater than 1 cm, an avascular wall of less than 1 mm, and soft margins [1]. A giant emphysematous bulla (GEB), which is also called vanishing lung syndrome, refers to cases in which blisters account for more than 30% of the hemithorax [2]. Patients with GEBs are at risk of potential complications from blisters during surgery. Pneumothorax emphysema, atelectasis, and fatal cardiovascular collapse might occur during surgery if these patients are given positive pressure ventilation. If a GEB is detected in a preoperative examination, it is recommended to proceed with the operation while maintaining spontaneous breathing if possible [3]. However, if an operation has to be performed without maintaining spontaneous breathing, the operation should proceed in preparation for cardiovascular collapse due to the rupturing of the bulla or the mass effect of giant bullae before and after the operation. Here, we report a case of cardiovascular collapse due to the mass effect of giant bullae during non-thoracic emergency surgery in a patient with a GEB.

## 2. Case Report

A 43-year-old man (height: 173 cm, weight: 75 kg) was admitted to the emergency room with a stab wound to the abdomen after being stabbed by falling over an iron rod that came out of a fence. It was removed when the patient came to the emergency room. There was a 3 cm stab wound in the upper-right quadrant of the patient’s abdomen, and the peritoneum was open. The patient’s trauma was only a stab wound confined to the abdomen, and chest trauma was not found.

**Figure 1:**
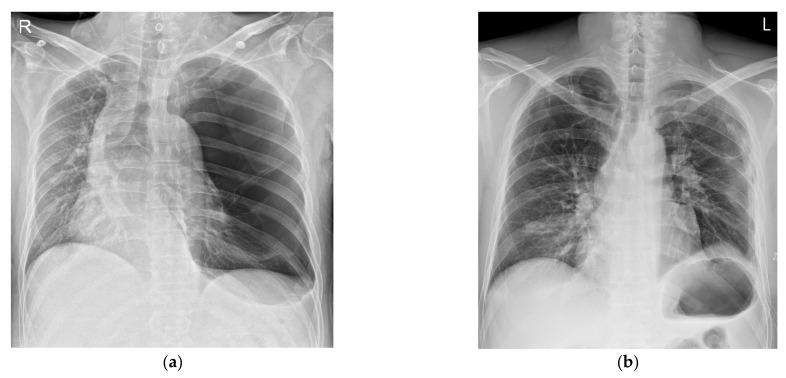
Chest X-ray at the time of admission (**a**) and on the first day after the operation (**b**). It can be seen that the left lung collapsed, and the mediastinum shifted to the right (**a**).

**Figure 2:**
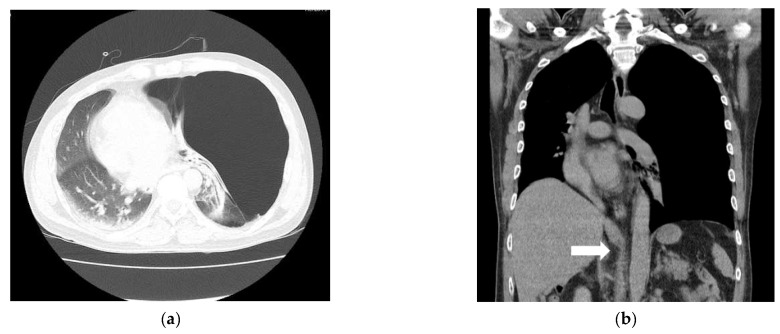
Giant bullae caused a mass effect that deviated the mediastinum (**a**). The inferior vena cava (IVC) was bent due to the mediastinal shift (white arrow) (**b**).

Other diseases and surgical history were denied. His vital signs in the emergency room were stable with a blood pressure (BP) of 145/90 mmHg, a heart rate (HR) of 89 bpm, a respiratory rate of 20 per minute, a SpO_2_ level of 97%, and a body temperature of 36 °C. An abdominal CT scan showed active bleeding in the right rectus sheath muscle and hemoperitoneum. Thus, emergency surgery was decided upon. There were no abnormal preoperative laboratory findings or electrocardiographic findings. A chest X-ray showed a large radiolucent area of the left lung with contralateral mediastinal deviation, so the emergency medicine physician suspected a pneumothorax. The pneumothorax seen on the X-ray was large, but the patient had no pneumothorax symptoms, such as dyspnea or chest pain. Therefore, a chest CT was performed. However, the CT showed a GEB in the left lung and bullous emphysema in the right lung with displacement of the mediastinal structures (Figure 1a and Figure 2a). In cases with GEBs, unlike with a pneumothorax, chest tube drainage (CTD) is not placed because the bullae could be damaged and tension pneumothorax could occur. Although the patient had no symptoms of dyspnea, it was decided to remove the GEB first and then perform abdominal surgery in consultation with a thoracic surgeon, since the GEB could affect the patient’s vital signs during positive pressure ventilation.

In GEBs, a pneumothorax tends to occur when positive pressure ventilation is performed. The incidence of tension pneumothorax is high [4]. Therefore, we planned anesthesia to avoid positive pressure ventilation as much as possible. It was decided to use succinylcholine to supply 100% oxygen for at least 3 min before the induction of anesthesia and to provide as little positive pressure ventilation as possible before intubation. After endotracheal intubation of the double-lumen tube, it was decided to exclude the use of non-depolarizing muscle relaxants and positive pressure ventilation until the tube position was confirmed. When the effect of succinylcholine disappeared and spontaneous breathing returned, spontaneous breathing was planned to be maintained until one-lung ventilation was started. Before the induction of anesthesia, the patient’s vital signs were relatively normal, with a BP of 123/85 mmHg, an HR of 100 bpm, and a SpO_2_ level of 100%.

After preoxygenation for 3 min before the scheduled induction of anesthesia, total intravenous anesthesia (TIVA: target concentration, propofol: 4 mg/mL, remifentanil: 4 ng/mL) with propofol and remifentanil was started. For endotracheal intubation, 70 mg of succinylcholine was administered. Patients with GEBs may experience rapid hemodynamic changes during induction of anesthesia and positive pressure ventilation. Therefore, anesthesia was performed with succinylcholine so that the patient could quickly recover to spontaneous breathing in the case of a hemodynamically dangerous situation.

Immediately after endotracheal intubation with a 37Fr double-lumen tube (DLT), the patient’s vital signs were normal with a BP of 115/80 mmHg, an HR of 80 bpm, and a SpO_2_ level of 98%. The location of the DLT was confirmed by using a flexible bronchoscope without positive pressure ventilation. However, shortly thereafter, the patient’s BP and HR dropped to 46/27 mmHg and 30 bpm. His oxygen saturation was 94%. We immediately administered 10 mg of ephedrine twice and stopped the drugs that were administered with TIVA. However, his BP and HR did not improve. Considering the possibility of tension pneumothorax, after the administration of 50 mg of rocuronium, right one-lung ventilation was immediately performed. After consulting with a thoracic surgeon, we decided to quickly check the condition of the bulla via thoracoscopy. Dobutamine at 10 mg/kg/min was started before surgery, and sufficient fluid administration was continued.

During the thoracoscopic surgery, the thoracic surgeon confirmed that the GEB had not ruptured. His BP and HR returned to normal ranges after the operation began. A wedge resection was performed on the apical and lingular segments of the upper-left lobe. His BP and HR were within the normal ranges during the operation.

After the bullectomy, the supine position was changed, and surgery was performed for the abdominal wound area. For the surgical operation, laparotomy was performed. His vital signs were stable during the abdominal surgery. The patient recovered from anesthesia with stable vital signs. The total time under anesthesia was 245 min, and the operation’s time was 215 min. The patient was transferred to the intensive care unit, where his vital signs remained stable. The patient was transferred from the intensive care unit to the general ward after 16 h. A chest X-ray taken on the second day after the surgery showed no special findings (Figure 1b). The chest tube drainage was removed on the 8th day after the operation. The patient was discharged without complications on the 12th day after the operation.

Ethical approval was not required for this case report because no interventions or changes were made in the clinical course of events. However, written informed consent was obtained from the patient.

## 3. Discussion

A lung bulla is an air cyst with a diameter greater than 1 cm when inflated. As stated previously, a GEB can be defined when a bulla occupies more than 30% of the hemithorax [2]. It has been reported that GEBs occur frequently in young men [5]. They are also accompanied by diseases such as Marfan syndrome, Ehlers–Danlos syndrome, and alpha-1 antitrypsin deficiency. Acquired factors may include smoking and marijuana use. The patient had never smoked cigarettes or marijuana and had not been exposed to smoke occupationally. GEBs are also related to lung infection, chronic obstructive pulmonary disease, mechanical ventilation, and so on [2,6]. They mainly occur in the upper lobe. Their clinical pictures are often asymptomatic. However, they can be accompanied by coughing, shortness of breath, and chest pain.

Regarding the pathophysiology of bullous disease, a bulla of the lung is caused by the destruction of the parenchyma by preventing gas from entering but not leaving the cystic space due to valvular obstruction. Due to the continuous destruction of the alveoli and the effect of the expansion of the gas entering the space, the space steadily expands, causing the surrounding lungs to be compressed and collapse. This is conceptually called an intrapulmonary pneumothorax [7]. The cause of cardiovascular collapse is presumed to be cardiorespiratory physiomechanics due to giant bullae [6].

For the diagnosis of a GEB, a chest X-ray is primarily performed. X-ray findings have shown large bullae that account for one-third of the hemithorax, lung parenchyma atelectasis, inverted ipsilateral diaphragm, and contralateral displacement of the mediastinum [8]. GEBs and pneumothorax are different in chest X-rays. In the chest CT scan, there was no pneumothorax, but giant bullae were shown over the entire lung. In the case of a GEB, the entire parenchyma is compressed downward in the direction of the costal angle, while a pneumothorax appears as a lung parenchyma that collapses into a clump toward the hilum [9]. However, plain chest X-rays and physical examinations cannot provide accurate anatomical information on the findings. In addition, if a CTD is mistakenly inserted for a pneumothorax, the GEB, which has a very thin wall compared to the normal lung parenchyma, is easily damaged and can lead to a dangerous situation, such as tension pneumothorax. Therefore, chest CT is more appropriate as a test for differentiating GEBs [10]. CT can also be a good test, as it can diagnose other coexisting diseases. In the present case, the left lung was radiolucent on chest PA, and the mediastinum was deviated to the right, suggesting tension pneumothorax (Figure 1a). In the chest CT scans, it was revealed that it was not a pneumothorax, as giant bullae were shown over the entire lung. These giant bullae caused a mass effect that deviated the mediastinum (Figure 2a). In addition, it could be seen that the inferior vena cava (IVC) was bent due to the mediastinal shift in the coronal plane of the chest CT (Figure 2b). This can lead to cardiovascular collapse due to the mass effect of the GEB itself, as well as tension pneumothorax due to the rupturing of the GEB. We speculate that this mass effect might have been exaggerated by muscle relaxation after succinylcholine use. Superior vena cava syndrome caused by a GEB pressing the superior vena cava has been reported [11]. However, cases showing IVC-syndrome-like symptoms by pressing the IVC have not yet been reported.

In addition, in the case of a huge bulla such as that described here, it might be difficult to differentiate it from a pneumothorax. This patient’s chest X-ray was also initially read as a pneumothorax. It is known that tension pneumothorax can be life-threatening. Thus, treatment should not be delayed while waiting for radiological confirmation [12]. However, if a huge bulla is mistaken for a pneumothorax and CTD is placed, care must be taken, as an uncontrolled pneumothorax or air fistula might occur due to iatrogenicity [13]. If the GEB is ruptured during general anesthesia, tension pneumothorax might occur, and the patient might fall into a more dangerous condition. Several efforts are required to avoid this situation. First, nitrous oxide should not be used during anesthesia. Second, the peak inspiratory pressure should be kept below 20 cm H_2_O with the tidal volume set to 5–6 mL/kg of ideal body weight. Third, hypercapnia should be allowed, and the I:E ratio should be maintained at around 1:4–1:5 to prevent air trapping. Finally, early extubation should be attempted [3].

## 4. Conclusions

GEB can cause several cardiopulmonary complications. In particular, tension pneumothorax caused by the rupturing of bullae can lead to life-threatening hypoxia or decreased cardiac output. Hemodynamic instability due to the mass effect of bullae might also occur, as in this case. Thus, treatment should be performed with thorough preparation for anesthesia and surgery for patients with giant bullae.

## Data Availability

Not applicable.
